# Implantation of a sutureless aortic valve into the ring of a mechanical valve: an extreme solution for extreme cases

**DOI:** 10.1093/icvts/ivae207

**Published:** 2024-12-14

**Authors:** Robič Boris, Rene Petrovič, Arta Krasniqi, Miha Antonič

**Affiliations:** Clinical Department for Cardiac Surgery, University Medical Centre Maribor, Maribor, Slovenia; Clinical Department for Cardiac Surgery, University Medical Centre Maribor, Maribor, Slovenia; Clinical Department for Cardiac Surgery, University Medical Centre Maribor, Maribor, Slovenia; Clinical Department for Cardiac Surgery, University Medical Centre Maribor, Maribor, Slovenia

**Keywords:** Aortic valve stenosis, Thrombosis, Structural valve deterioration, Sutureless aortic valve implantation, Cardiopulmonary bypass

## Abstract

Reoperations due to dysfunction of artificial mechanical aortic valves represent a complex and high-risk surgical procedure, particularly for elderly patients. In this report, we present a case of an 81-year-old female patient where, due to structural degeneration of the mechanical valve, an emergency surgical procedure was indicated. After the removal of the valve leaflets, a sutureless aortic valve was implanted within the mechanical ring. In our case, we demonstrate that such an approach is a safe alternative for high-risk patients, who cannot undergo percutaneous procedures. By reducing the duration of cardiopulmonary bypass and aortic clamping, the surgical outcomes and complication rates can be significantly improved.

## INTRODUCTION

Reoperations for artificial mechanical aortic valve dysfunction are associated with high mortality due to prolonged cardiopulmonary bypass, aortic cross-clamping and the need for complex reconstructive interventions. This risk is even higher for the elderly. We present a case where the use of a sutureless valve implanted within the ring of a mechanical valve helped minimize the aortic cross-clamp time and avoid a potentially complex aortic root reconstruction.

## CASE PRESENTATION

**Figure 1:**
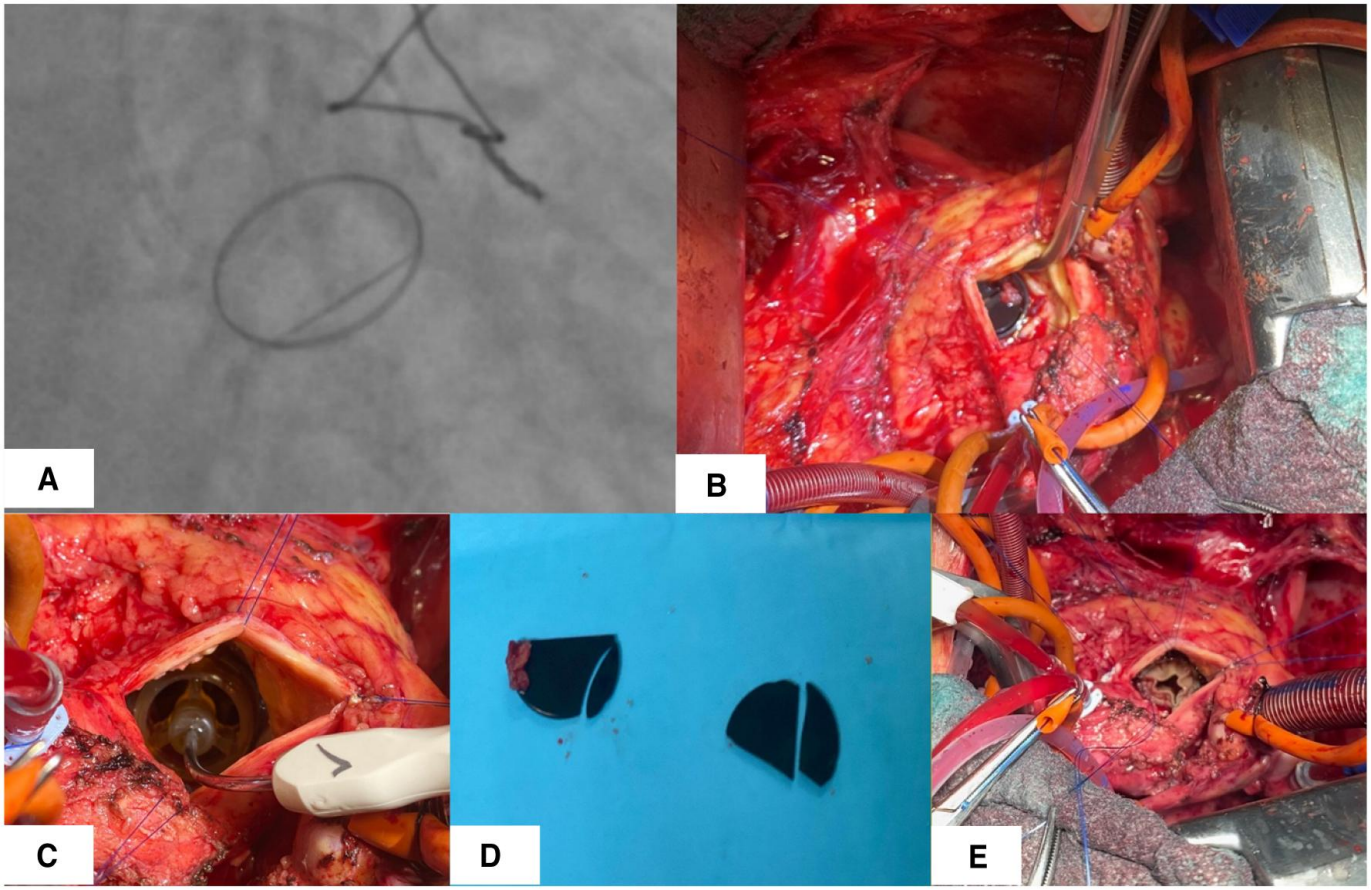
(**A**) Obstructed artificial aortic valve leaflet during invasive cardiac diagnostics. (**B**) Formation of a thrombus obstructing the leaflet of a mechanical aortic valve. (**C**) Insertion of a Perceval size S sizing gauge through the ring of the mechanical valve. (**D**) Leaflets and fragments of the removed mechanical valve. (**E**) Final position of the implanted sutureless valve within the ring of the mechanical valve.

An 81-year-old female, with a history of aortic valve replacement with a mechanical prosthesis 20 years ago, atrial fibrillation, arterial hypertension, hyperlipidaemia and hypothyroidism presented to our clinic with progressive dyspnoea. Diagnostic workup revealed severe aortic stenosis, moderate aortic regurgitation and impaired mobility of one of the leaflets of the mechanical aortic valve (Fig. 1A). Due to deteriorating clinical status, we opted for urgent surgery. Given the patient’s overall poor condition, our plan aimed at minimizing aortic cross-clamping and cardiopulmonary bypass time.

After induction of general anaesthesia, a median sternotomy incision was made along the previous scar. Since the patient had undergone surgery through a ministernotomy in 2003, significant adhesions were only present around the ascending aorta, right atrium and right upper pulmonary vein. After adhesiolysis and central cannulation, cardiopulmonary bypass was initiated. Following aortic cross-clamping and cardioplegia delivery, an aortotomy was performed distally to the previous suture line. Valve inspection revealed formed thrombi obstructing the leaflet of the mechanical aortic valve (Fig. 1B).

**Figure 2:**
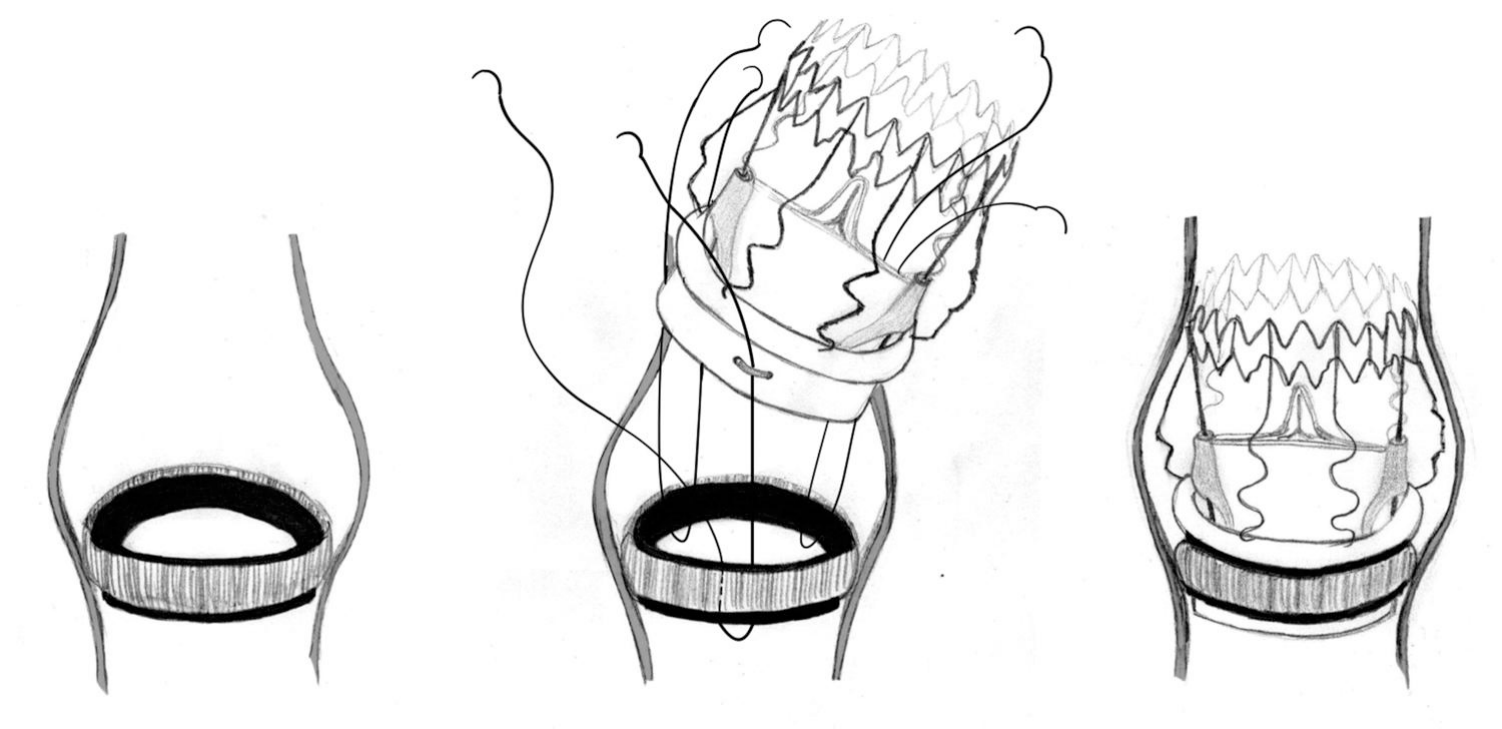
An illustration depicting the insertion of a sutureless aortic valve within the framework of a mechanical aortic valve.

Considering the potential for prolonged operative time and the associated risk of necessitating a complex aortic root reconstruction, the decision was made to pursue a surgical approach removing only the mechanical valve leaflets while maintaining the integrity of the valve ring. Subsequently, a sutureless aortic valve was implanted into the retained ring of the mechanical valve, as depicted in Fig. 1C. A small, folded cloth was inserted into the left ventricle to catch debris and potential fragments, which was later removed (Fig. 1D). Following the removal of the leaflets, guiding sutures were carefully positioned at the lowest point of each aortic valve cusp to facilitate valve placement. Subsequently, a Sorin Perceval size S sutureless aortic valve was implanted, as depicted in Fig. 1E. The choice of Sorin Perceval size S was determined based on the manufacturer’s specifications, which indicated compatibility with the SJM Aortic Mechanical valve size 23. To enhance stability, the guiding sutures, typically slated for removal, were securely tied. The implanted valve underwent post-dilation using a balloon inflated to 2 atmospheres for 30 s (Fig. 2). Following aortic closure, the patient was successfully weaned off cardiopulmonary bypass. Intraoperative transoesophageal echocardiography confirmed optimal valve functionality without regurgitation. The duration of aortic cross-clamping was 44 min, with a total cardiopulmonary bypass time of 74 min. The patient’s postoperative course was prolonged due to a sternal wound infection, but she was eventually discharged home. At the follow-up checkup at 6 months, the patient reported she was feeling well and fit. The transthoracic echocardiogram showed excellent function of the artificial aortic valve with a maximum gradient of 26 mmHg and a mean gradient of 17 mmHg. The patient was switched to NOAC, and during the 6-month follow-up period, no thromboembolic complications were observed.

## DISCUSSION

In elderly patients aged over 80 necessitating aortic valve re-replacement, the prospect of reoperation poses considerable risks, with reported mortality rates in the literature soaring to 16.4% [1]. Guidelines underscore the preference for transcatheter aortic valve replacement over surgical aortic valve replacement in patients aged over 75 [2], with a notable exception made for individuals with pre-existing mechanical aortic valve implants. A case documented by Butter *et al.* [3], however, showcased a successful transcatheter aortic valve replacement in a patient with a mechanical prosthesis. To the best of our knowledge, our case marks the 1st instance of sutureless aortic valve implantation within the confines of a mechanical valve ring. This approach eliminates the need for the labour-intensive and time-consuming removal of the fused ring from the aortic wall, a process that might also have necessitated extensive aortic root reconstructive procedures. The main advantage of this technique lies in its potential to streamline the procedure that could significantly reduce the duration of both cardiopulmonary bypass and aortic cross-clamp. Reoperations inherently entail prolonged durations of cardiopulmonary bypass and aortic cross-clamping, factors known to independently predict mortality and morbidity, contributing to diminished cardiac output, prolonged mechanical ventilation, renal impairment and neurological deficits [4]. Encouraging outcomes were reported by Cavozza *et al.* [5], who demonstrated favourable results with abbreviated aortic cross-clamping times during Edwards Intuity valve implantation within a mechanical valve ring, mirroring our approach.

## CONCLUSION

In this clinical case, we demonstrate that the implantation of a sutureless valve within the ring of a mechanical valve can be a safe alternative for high-risk patients. This is especially crucial in cases where percutaneous techniques are not applicable, and where shortening the duration of cardiopulmonary bypass and aortic cross-clamping is crucial. Another critical consideration is the management of the patient’s anticoagulant therapy, for which further studies and longer patient follow-up are warranted.

## Data Availability

The data underlying this article are available in the article and in its online supplementary material. All included Figures have been created by the authors.
